# Role of Autotaxin in the Pathogenesis of Retina Ischemia and Its Therapeutic Implications

**DOI:** 10.3390/ijms27062776

**Published:** 2026-03-19

**Authors:** Ryo Terao, Ryosuke Fujino, Kentaro Hayashi, Takafumi Suzuki, Shota Shimizu, Reiko Yamagishi, Takashi Ueta, Tomoyasu Shiraya, Megumi Honjo, Makoto Aihara

**Affiliations:** 1 Department of Ophthalmology, Graduate School of Medicine, The University of Tokyo, Tokyo 113-8655, Japan; 2 Department of Ophthalmology, Showa General Hospital, Tokyo 187-8510, Japan

**Keywords:** retinal vein occlusion, macular edema, autotaxin

## Abstract

Retinal vein occlusion (RVO) is a common vascular disease that leads to vision loss due to macular edema (ME). This study investigated the role of autotaxin (ATX), a lysophospholipase D, in the pathogenesis of RVO. In mice, RVO was induced by intravenous administration of rose bengal followed by laser irradiation of retinal veins. ATX expression in the retina was evaluated using immunohistochemistry. Intravitreal ATX was administered, and retinal changes were assessed using fluorescence angiography and optical coherence tomography (OCT). In human retinal microvascular endothelial cells (HRMECs), intercellular barrier function was evaluated using transepithelial electrical resistance (TEER). In the murine RVO model, the ATX inhibitor HA130 was administered intravitreally, and retinal thickness was measured and compared using OCT. ATX expression was increased in retinal vessels in the RVO model. Intravitreal administration of ATX induced retinal edema and serous retinal detachment (SRD). ATX significantly disrupted the barrier integrity of HRMECs and promoted the expression of vascular endothelial growth factor (VEGF), which was ameliorated by HA130. Intravitreal administration of HA130 significantly reduced retinal thickening caused by retinal edema secondary to RVO and the elevated expression of intercellular adhesion molecule (ICAM)-1 in the retina. These findings suggest that ATX plays a critical role in RVO-induced ME by disrupting endothelial barrier integrity, potentially through the upregulation of VEGF in retinal endothelial cells and subsequent ICAM-1 upregulation in the retina.

## 1. Introduction

Retinal vein occlusion (RVO), a prevalent vascular disease characterized by retinal hemorrhage and dilated veins, significantly affects the vision of millions of people worldwide [1,2,3]. At the molecular level, vascular endothelial growth factor (VEGF), interleukins (ILs), and monocyte chemoattractant protein-1 (MCP-1) are implicated in the pathogenesis of RVO and the development of macular edema (ME) [4,5]. ME, a leading cause of vision impairment, is a common complication of retinal ischemic diseases such as RVO [6]. Its causes are multifactorial, including inflammation, increased retinal vascular permeability, and ischemia [7]. During RVO, the primary factor in ME development is disruption of the inner blood retinal barrier (BRB). The BRB is formed by tight junctions between retinal capillary endothelial cells and retinal pigment epithelial (RPE) cells, which constitute the inner and outer retinal layers, respectively [8]. The BRB regulates vascular permeability and plays an essential role in retinal fluid clearance [9,10]. In retinal vascular diseases such as RVO, increased levels of inflammatory mediators, such as VEGF, contribute to BRB disruption [7]. Intravitreal injection of anti-VEGF agents is currently the first-line treatment for ME secondary to RVO, with efficacy demonstrated in large clinical studies [11,12,13,14]. However, a 5-year follow-up study of patients with RVO treated with anti-VEGF therapy reported that 35% still had a best-corrected visual acuity (BCVA; logMAR) of 0.3 or higher. Furthermore, ME secondary to RVO required frequent anti-VEGF injections, with a cumulative total of 28–29 injections over 5 years [15], imposing a substantial treatment burden on both patients and healthcare providers. These findings highlight the limitations of current treatments, necessitating the exploration of new therapeutic strategies beyond anti-VEGF therapy.

Autotaxin (ATX), a lysophospholipase D (lysoPLD), converts lysophosphatidylcholine (LPC) into lysophosphatidic acid (LPA), which acts on G-protein-coupled receptors to induce cellular responses such as proliferation, migration, and contraction [16,17]. The ATX/LPA pathway has been implicated in various pathologies, including rheumatoid arthritis [18] and dexamethasone-induced fibrotic responses in human trabecular meshwork cells [19,20,21]. In retinal diseases, a clinical study found significantly elevated ATX and LPA levels in the vitreous fluid of patients with RVO, which were positively correlated with proinflammatory cytokines and VEGF-A [22]. This suggests a potential role of ATX in the pathogenesis of RVO; however, its precise contribution remains unclear.

Therefore, we investigated the role of ATX in the pathogenesis of RVO using a murine RVO model that recapitulates key features of human RVO, including characteristic fundus appearances and histologically confirmed retinal edema consistent with clinical observations [23]. We also examined the effects of ATX inhibition in RVO to identify potential new therapeutic interventions.

## 2. Results

### 2.1. ATX Expression in Retinal Vasculature in the Murine RVO Model

**Figure 1:**
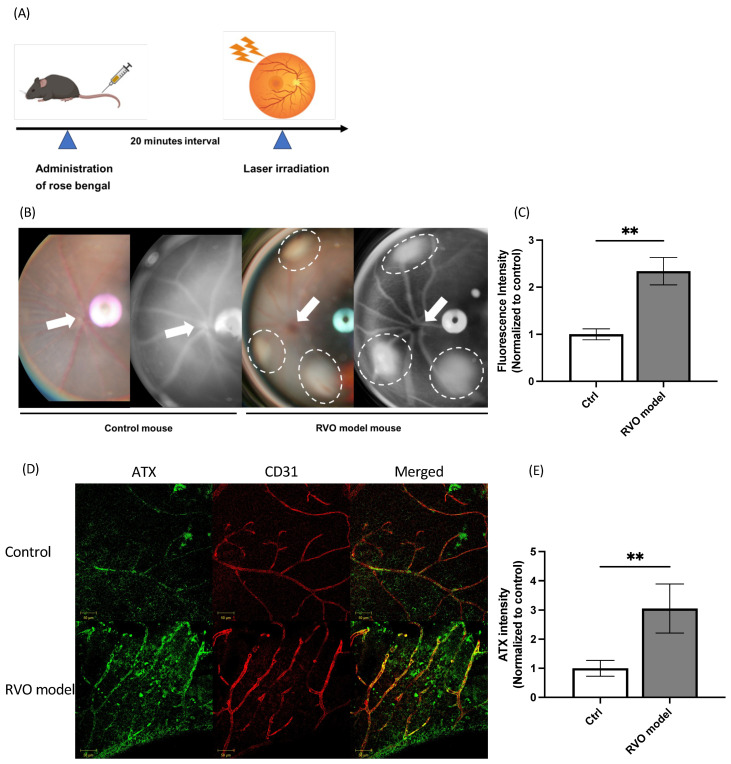
Generation of the RVO model and expression of autotaxin in the retina. (**A**) Experimental design of RVO model induction. Rose bengal (0.15 mL) was injected into the penile vein. After 20 min, the branch veins were irradiated with a laser. (**B**) Fundus photography and fluorescence angiography (FA) of the RVO model. Fundus photography (far left) and FA (second from the left) images of the control eye show an intact retina. In the fundus (second from the right) and FA (far right) images of the RVO model, three retinal veins are occluded (white dotted circles), and fluorescence leakage from the surrounding vessels is observed. White arrows indicate the optic disc. (**C**) Quantification of fluorescence intensity in the control retina and RVO retina. *n* = 4. (**D**) Immunofluorescence images of retinal flat mounts isolated from control and RVO models. In the control retina, ATX expression in retinal vessels colocalized with CD31, a marker for vascular endothelial cells. ATX expression is increased in the retina of the RVO model. (**E**) Quantification of fluorescence intensity in vessels of control and RVO retinas. Control, *n* = 8; RVO model, *n* = 10. ** *p* < 0.01. Scale bar: 50 µm. RVO, Retinal vein occlusion; ATX, Autotaxin.

The murine RVO model was generated and confirmed by a significant increase in fluorescence intensity in the retinal vessels (Figure 1A–C, 1.0 ± 0.12 vs. 2.4 ± 0.29; *p* = 0.0079). To evaluate ATX expression in the retina in the RVO model, immunofluorescence analysis was performed using retinal flat mounts. The control retina showed ATX expression in retinal vessels. Furthermore, ATX expression was significantly increased in the retina of the RVO model (Figure 1D,E; 1.0 ± 0.27 vs. 3.1 ± 0.84; *p* = 0.0079). These results indicate that ATX is expressed in the retinal vascular endothelial cells, and its expression is upregulated following RVO induction.

### 2.2. ATX Induces Retinal Edema

**Figure 2:**
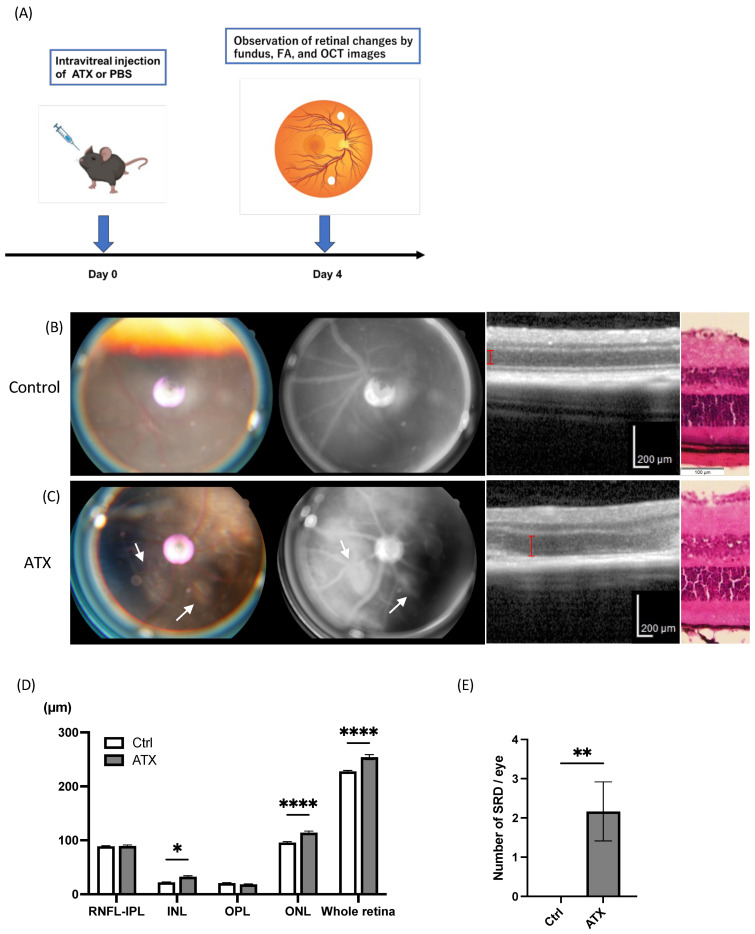
(**A**) Experimental design of intravitreal autotaxin (ATX) administration. (**B**) Fundus photography, fluorescence angiography (FA), optical coherence tomography (OCT) images, and hematoxylin-eosin (H & E) staining of the eye treated with phosphate-buffered saline (PBS). No retinal edema was observed. (**C**) Retinal changes in a mouse treated with ATX. Representative fundus photography and FA images show fluorescence leakage. Arrows indicate fluorescein leakage from retinal vessels following ATX administration. Bars indicate outer nuclear layer thickness. (**D**) Quantification of retinal thickness after ATX administration. *n* = 6; two-way analysis of variance followed by Bonferroni post hoc test. (**E**) Number of serous retinal detachments (SRDs) after ATX administration. *n* = 6; Wilcoxon rank sum test. * *p* < 0.05, ** *p* < 0.01, **** *p* < 0.0001.

Since ATX expression was increased in the RVO model, we next examined the biological effects of ATX on retinal phenotypes (Figure 2A). Based on the reported physiological concentration of serum ATX (5–10 µM) [24], 100 μM ATX (1.5 µL) was administered intravitreally, which corresponds to approximately 20 µM in the vitreous. As a result, eyes treated with ATX developed retinal vascular leakage and edema (Figure 2B,C). OCT analysis revealed retinal thickening, particularly in the inner nuclear layer and outer nuclear layer (22.1 ± 2.78 vs. 32.3 ± 7.82, 95.9 ± 5.35 vs. 114 ± 10.9, *p* = 0.0152, <0.0001, respectively; Figure 2D). In addition, ATX induced serous retinal detachments (SRDs) (0 ± 0 vs. 2.2 ± 0.75, *p* = 0.0022; Figure 2E). These findings suggest that ATX induces retinal vascular leakage, leading to retinal edema.

### 2.3. ATX Disrupts Cellular Barrier Function and Promotes VEGF in Retinal Vascular Endothelial Cells

**Figure 3:**
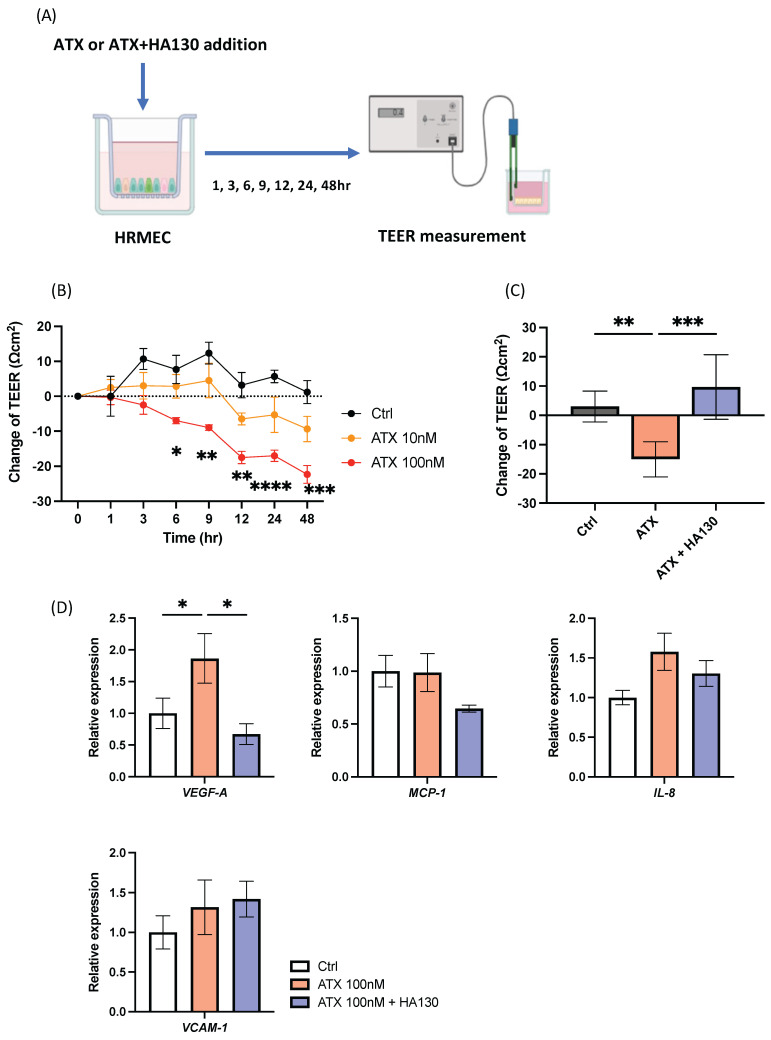
(**A**) Experimental design of transepithelial electrical resistance (TEER) measurement in primary human retinal microvascular endothelial cells (HRMECs) treated with autotaxin (ATX) and/or the ATX inhibitor HA130. (**B**) Time−course changes in TEER in HRMECs treated with ATX (100 nM or 10 nM) or phosphate−buffered saline (PBS; control). (**C**) Effects of the ATX inhibitor HA130 (100 nM) on ATX−induced barrier dysfunction in HRMECs. (**D**) mRNA expression of cytokines in HRMECs, evaluated by quantitative polymerase chain reaction (qPCR). ATX significantly reduces endothelial barrier integrity and increases VEGF expression, both of which are suppressed by HA130. *n* = 6; two−way analysis of variance followed by Dunnett’s test for (**B**) and one−way analysis of variance followed by Tukey−Kramer test for (**C**,**D**). Error bars indicate standard deviation. * *p* < 0.05, ** *p* < 0.01, *** *p* < 0.001, **** *p* < 0.0001. VEGF−A, vascular endothelial growth factor−A; MCP−1, monocyte chemotactic protein−1; IL, interleukin; VCAM−1, vascular cell adhesion molecule−1.

Based on the findings that ATX induced fluid leakage from retinal vasculature, we next assessed the barrier function of retinal microvascular endothelial cells (HRMECs). Transepithelial electrical resistance (TEER), which reflects cellular barrier integrity, was measured to evaluate changes in endothelial barrier function over time (Figure 3A). TEER values significantly decreased beginning at 6 h after treatment with 100 nM ATX, suggesting that ATX disrupts HRMEC barrier function (Figure 3B). At 48 h, the change in TEER decreased from 1.2 ± 8.2 Ω·cm^2^ (control) to −22.3 ± 6.3 Ω·cm^2^ (100 nM ATX; *p* = 0.0005).

To investigate whether ATX inhibition protects endothelial barrier integrity, TEER was measured in HRMECs treated with the ATX inhibitor HA130 (100 nM), which reduces the lysoPLD activity of ATX to convert LPC into LPA, together with 100 nM. HA130 significantly suppressed the ATX-induced decrease in TEER (control 3.0 ± 5.3, ATX −13.4 ± 3.1, ATX + HA130 12.6 ± 9.4; ATX vs. ATX + HA130; *p* = 0.0002; Figure 3C). A similar reduction in barrier integrity was also observed in human umbilical vein endothelial cells (HUVECs) (Supplementary Figure S1). Furthermore, 100 nM ATX significantly increased *VEGF-A* mRNA expression, which was suppressed by HA130 (ATX vs. ATX + HA130, *p* = 0.0149; Figure 3D). These results indicate that ATX disrupts retinal endothelial barrier function and promotes VEGF-A expression, both of which are suppressed by ATX inhibition.

### 2.4. ATX Inhibition Suppresses Retinal Edema in the Murine RVO Model

**Figure 4:**
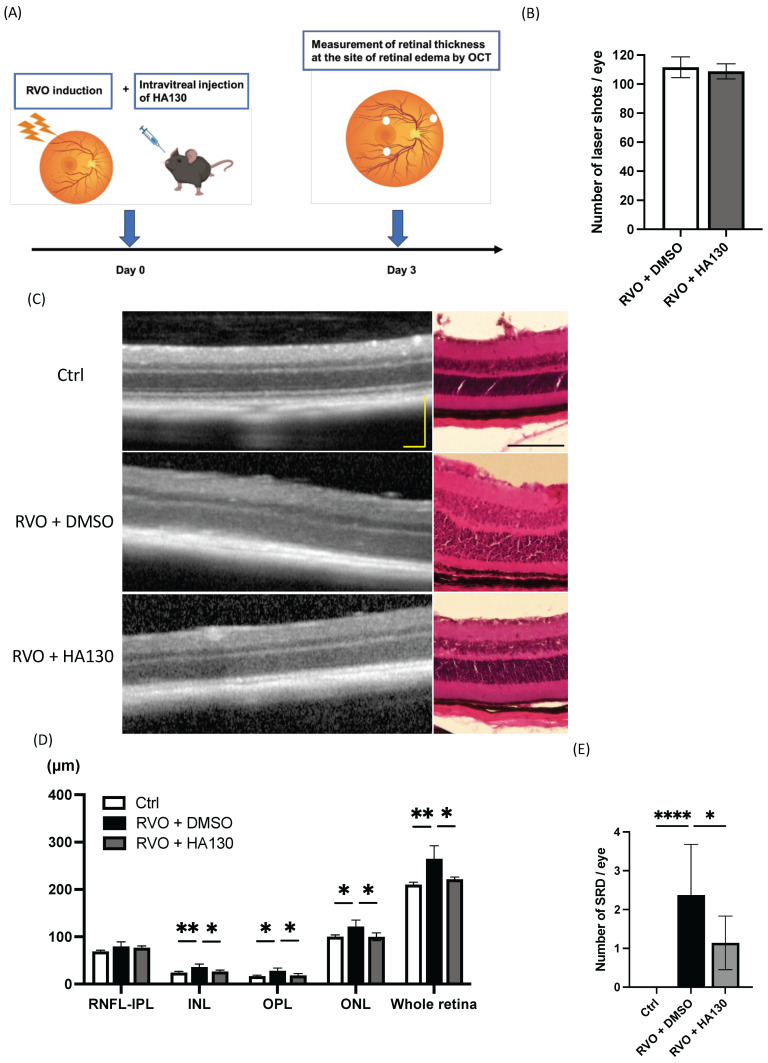
(**A**) Experimental design of RVO model induction and intravitreal administration of the ATX inhibitor HA130 for retinal thickness measurement. (**B**) Comparison of the number of laser shots between experimental groups. (**C**) Representative images of control eyes, RVO eyes treated with vehicle (DMSO), and RVO eyes treated with HA130. (**D**) Quantification of retinal thickness measured by OCT. (**E**) Quantification of the number of serous retinal detachments (SRDs). *n* = 6. One-way analysis of variance followed by Tukey–Kramer test for (**D**,**E**). Error bars indicate standard deviation. * *p* < 0.05, ** *p* < 0.01, **** *p* < 0.0001. RVO, Retinal vein occlusion; HA130, autotaxin inhibitor; OCT, optical coherence tomography; DMSO, dimethyl sulfoxide; RNFL, retinal nerve fiber layer; INL, inner nuclear layer; OPL, outer plexiform layer; ONL, outer nuclear layer. Scale bar = 200 μm.

Based on the findings of this study, ATX expression is upregulated in retinal vessels in RVO and disrupts vascular endothelial barrier integrity, thereby increasing vascular permeability and inducing retinal edema. Therefore, we investigated the therapeutic effect of the ATX inhibitor HA130 on retinal edema in the murine RVO model by measuring retinal thickness (Figure 4A).

**Figure 5:**
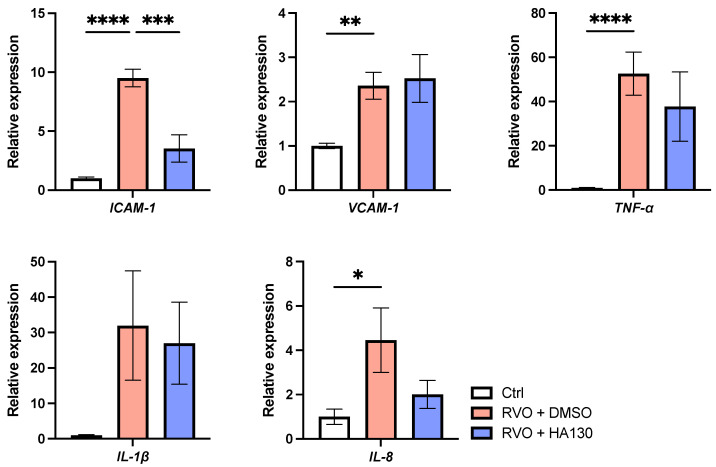
mRNA expression of inflammatory cytokines in the retina of control, RVO, and RVO + HA130 groups, evaluated by qPCR. HA130 suppressed the expression of ICAM-1, which was upregulated in the RVO retina. *n* = 6. One-way analysis of variance followed by Tukey–Kramer test. * *p* < 0.05, ** *p* < 0.01, *** *p* < 0.001, **** *p* < 0.0001. RVO, retinal vein occlusion; DMSO, dimethyl sulfoxide; ICAM-1, intercellular adhesion molecule-1; VCAM-1, vascular cell adhesion molecule-1; TNF-α, tumor necrosis factor-α; IL, interleukin. *n* = 6.

Although DMSO was used as a vehicle for HA130 administration, no retinal structural abnormalities or vascular leakage were observed in vehicle-treated eyes, suggesting that the observed effects were not attributable to vehicle toxicity. There was no significant difference in the number of laser shots between the RVO group and the RVO model + HA130 group (112 ± 7.2 vs. 109 ± 5.2; Figure 4B). However, retinal thickness in the RVO model was significantly reduced by HA130 administration (210.1 ± 5.12 vs. 264.9 ± 27.3 vs. 221.4 ± 4.77 μm; Figure 4C,D). HA130 also significantly reduced the number of SRDs in the RVO model (0 ± 0 vs 2.4 ± 1.3 vs 1.1 ± 0.69; Figure 4E). Moreover, HA130 suppressed the expression of intercellular adhesion molecule-1 (*ICAM-1*), which was upregulated in RVO retina (Figure 5). These findings suggest that ATX inhibition attenuates retinal edema, possibly through suppression of inflammatory signaling in the RVO retina.

## 3. Discussion

The aim of the present study was to investigate the role of ATX/LPA in the pathogenesis of RVO and to assess its potential as a new therapeutic target. In the retina, ATX was expressed in the retinal vascular endothelial cells, and its expression was increased following induction of the RVO model. ATX also induced leakage from retinal vessels in vivo. In vitro, ATX reduced endothelial barrier integrity and increased VEGF-A expression in retinal vascular endothelial cells, both of which were suppressed by the ATX inhibitor HA130. These findings suggest that ATX may act as an upstream regulator of VEGF-mediated vascular dysfunction and inflammation in RVO. Additionally, HA130 ameliorated retinal edema and reduced the upregulation of ICAM-1 induced by the RVO model. Taken together, these findings highlight the potential of ATX inhibition as a therapeutic strategy for macular edema secondary to RVO. In the present study, we did not directly compare ATX inhibition with anti-VEGF agents because our primary aim was to explore the mechanistic role of ATX in RVO pathogenesis. However, our additional findings demonstrating that ATX upregulates VEGF expression in retinal endothelial cells suggest that ATX may function upstream of VEGF signaling. Therefore, ATX inhibition may represent a complementary or upstream therapeutic strategy rather than a direct alternative to anti-VEGF therapy. Future preclinical studies comparing efficacy, duration of action, and potential additive effects with anti-VEGF agents will be necessary to determine the translational significance of ATX inhibition.

In mammals, ATX plays a pivotal role in regulating endothelial cell functions, including cell migration, proliferation, and differentiation, which are essential for the control of vascular permeability. For example, ATX interacts with VEGF signaling and promotes vascular endothelial cell migration through activation of the LPA receptors [17]. ATX also plays an important role in the retina by promoting the contraction of retinal growth cone axons during retinal development [25,26]. ATX is expressed in retinal pigment epithelial (RPE) cells [27,28], and LPA has been shown to regulate BRB tight junctions in RPE cells [29]. While ATX is physiologically expressed predominantly in RPE cells, our study revealed that ATX expression was also present in retinal vascular endothelial cells. This finding is consistent with previous studies demonstrating ATX expression in vascular endothelial cells in other organs [30]. In fact, mice with endothelial cell-specific ATX deficiency exhibited reduced vascular permeability and smaller infarct sizes in cerebral vessels following ischemia–reperfusion while maintaining cerebral blood flow [31]. Upregulation of ATX has been reported to be driven by hypoxia and inflammation [32,33], both of which are important contributors to the development of RVO.

The increased expression of ATX in retinal vascular endothelial cells during RVO induction may contribute to the development of retinal edema. Under physiological conditions, the BRB prevents leakage of fluid from the vasculature into the neurosensory retina. However, in pathological conditions associated with macular edema, disruption of the BRB results in fluid accumulation in the macula, leading to retinal distortion and vision loss. In particular, disruption of the inner BRB, which is composed of retinal vascular endothelial cells, contributes to macular edema, including SRD, which are major causes of visual impairment in RVO and diabetic retinopathy [6,34,35]. Our in vitro experiments using HRMECs demonstrated that ATX disrupts endothelial barrier integrity. ATX plays a crucial role in regulating endothelial cell functions that control vascular permeability, including cell migration, proliferation, and differentiation [17]. Mechanistically, ATX interacts with VEGF-A signaling and promotes vascular endothelial cell migration through activation of LPA receptors [36]. Additionally, LPA generated by ATX increased permeability in mouse brain microvascular endothelial cells and decreased the expression of junction proteins such as β-catenin, VE-cadherin, and zonula occludens-1 [37]. These findings suggest that ATX plays a critical role in the regulation of endothelial barrier function in multiple tissues. Moreover, we found that the ATX inhibitor HA130 improved endothelial barrier integrity disrupted by ATX. ATX inhibitors have also been reported to suppress vascular endothelial permeability following ischemia–reperfusion in rat mesenteric capillaries [38], supporting the idea that ATX inhibition may reduce vascular leakage by restoring endothelial barrier function.

ATX generates LPA, a bioactive lipid that activates multiple downstream signaling pathways associated with inflammation and angiogenesis, including VEGF, VEGFR2, IL-6 and IL-8 [17,36,39]. LPA has been reported to promote VEGF-C expression through LPA1-3 receptors in prostate cancer cells [40]. Conversely, VEGF-A stimulates the expression of ATX and the LPA1 receptor in human umbilical vein cells [36]. The treatment with the ATX inhibitor BBT-877 has been shown to suppress mRNA levels of inflammatory cytokines such as IL-6 and MCP-1 in the kidneys of streptozotocin-induced diabetic mice [41]. Consistent with these findings, we observed that ATX increased VEGF-A in human retinal endothelial cells. Because VEGF-A, IL-6 and MCP-1 have been implicated in the pathogenesis of RVO [23], the therapeutic effects of ATX inhibition may partly result from suppression of vascular leakage and VEGF-A expression.

Another potential downstream pathway of ATX/LPA is the Rho/ROCK pathway. Previous studies have explored the therapeutic potential of Rho/ROCK inhibitors in RVO [17,42]. Rho/ROCK inhibition has been shown to improve retinal edema, nonperfusion areas, and retinal blood flow in mouse models of RVO by inhibiting phosphorylation of myosin phosphatase target subunit-1 (MYPT-1), a downstream effector of ROCK [42]. Although previous studies have implicated Rho/ROCK signaling as a downstream pathway of ATX/LPA signaling, we did not observe significant alterations in this pathway under our experimental conditions. This discrepancy may be explained by differences in experimental conditions, including the time points analyzed and the duration of RVO induction. It is also possible that ATX signaling in the context of RVO predominantly activates alternative pathways such as PI3K/Akt or MAPK/ERK, which have been shown to be downstream of LPA receptor signaling [43,44]. Further mechanistic studies are needed to clarify the relationship between ATX/LPA signaling and Rho/ROCK pathways in retinal vascular diseases.

In the present study, we further demonstrated the therapeutic potential of ATX inhibition for macular edema secondary to RVO. The significant reduction in retinal thickness in the RVO model following HA130 administration suggests that ATX inhibition may represent an effective strategy to reduce retinal edema. In addition, ATX inhibition reduced retinal ICAM-1 expression, suggesting that the ATX–LPA axis may contribute to endothelial activation and leukocyte adhesion in RVO. Because ICAM-1 is expressed not only in retinal endothelial cells but also in Müller glia and microglia under ischemic and inflammatory conditions [45] suppression of endothelial VEGF by HA130 may secondarily attenuate inflammatory activation of Müller glia. Therefore, the reduction in retinal ICAM-1 observed in vivo may reflect decreased glial activation resulting from suppressed endothelial VEGF signaling. These findings suggest that ATX inhibition may indirectly modulate retinal inflammation through an endothelial–glial signaling axis.

This study has several limitations. First, although we demonstrated the role of ATX using pharmacological modulation, genetic approaches such as endothelial cell-specific ATX knockout or inducible overexpression models would provide more definitive mechanistic insights. These models would help isolate the cell-type–specific contribution of ATX and validate our findings in a more physiologically relevant context. Second, only one ATX inhibitor was used in this study. Although HA130 has been widely used in previous studies because of its availability and specificity, additional validation using other ATX inhibitors such as PF-8380 (Selleck Chemicals, Houston, TX, USA), GLPG1690 (Galapagos NV, Mechelen, Belgium), S32826 (Cayman Chemical Company, Ann Arbor, MI, USA), and BBT-877 (Bridge Biotherapeutics, Inc., Seongnam, Republic of Korea) would help confirm the specificity of our findings. Third, a potential limitation of this study is that HA130 was dissolved in DMSO for intravitreal administration. Although DMSO may have potential ocular toxicity at high concentrations, no apparent retinal abnormalities were observed in the vehicle-treated eyes in our experiments. Nevertheless, the potential effects of DMSO should be considered when interpreting the results. Fourth, we did not directly investigate the cellular response of Müller glia, which may represent an important source of ICAM-1 in the RVO model. Because the present study focused on the ATX/LPA axis in retinal vascular endothelial cells, further studies should investigate the role of Müller glia in ATX-mediated retinal inflammation. Addressing these limitations will help further clarify the therapeutic relevance of ATX inhibition in retinal vascular diseases.

In summary, the present study identifies ATX as a key contributor to retinal vascular leakage and edema in RVO. These findings suggest that ATX acts upstream of VEGF signaling and contributes to endothelial barrier dysfunction in RVO, and that ATX inhibition may represent a potential new therapeutic strategy for retinal edema secondary to RVO.

## 4. Materials and Methods

### 4.1. Animals

All procedures were conducted in accordance with the Association for Research in Vision and Ophthalmology (ARVO) Statement for the Use of Animals in Ophthalmic and Vision Research. All experiments were approved by the Institutional Animal Research Committee of the University of Tokyo. Male C57BL/6J mice aged 8–10 weeks were purchased from Japan SLC (Hamamatsu, Japan) and housed in cages with free access to food and water under a standard 12 h light/dark cycle at 22 °C.

### 4.2. RVO Model

The murine RVO model was generated as previously described [23]. Mice were anesthetized with a mixture of ketamine (80 mg/kg; KETALAR, Daiichi Sankyo Propharma Co., Ltd., Tokyo, Japan) and xylazine (16 mg/kg; Selactar, Bayer, Leverkusen, Germany). Pupils were dilated with 1% tropicamide and 2.5% phenylephrine (Mydrin-P ophthalmic solution; Santen Pharmaceuticals Co., Ltd., Osaka, Japan), and SCOPISOL^®^ (Senju Pharmaceutical Co., Ltd., Osaka, Japan) was applied topically to the cornea to prevent desiccation. Rose bengal (8 mg/mL; Wako, Osaka, Japan) was administered via the penile vein (0.15 mL). Approximately 20 min after rose bengal administration, 30–40 laser shots per vein were applied to three branch veins per eye. A 532 nm diode laser (Novus Spectra; Lumenis Japan Co., Ltd., Tokyo, Japan) was used at 100–120 mW, with a duration of 300 ms and a spot size of 50 μm. Laser photocoagulation was applied approximately three disc diameters from the optic disc to avoid damaging other retinal vessels.

### 4.3. Immunofluorescence

Control mice and RVO model mice 1 day after the induction were euthanized by cervical dislocation under deep anesthesia, and their eyes were enucleated. The enucleated eyeballs were fixed in 4% paraformaldehyde for 30 min. Eyecups were dissected to prepare retinal flat mounts, followed by blocking with Blocking One Histo for 1 h. Samples were incubated overnight at 4 °C with primary antibodies (anti-Autotaxin; 1:50 dilution; Santa Cruz Biotechnology, Inc., Dallas, TX, USA, and anti-CD31; 1:200). After washing the retinal flat mounts with 0.3% Triton X-100 in phosphate-buffered saline (PBS), the samples were incubated for 1 h with Alexa Fluor-conjugated secondary antibodies (1:200; Thermo Fisher Scientific, Waltham, MA, USA) corresponding to the host species of the primary antibodies. Nuclei were stained with DAPI (4 µg/mL; Wako, Osaka, Japan). Samples were coverslipped with mounting medium. Images were captured using a confocal microscope (LSM880 with Airyscan; Carl Zeiss, Oberkochen, Germany).

### 4.4. Intravitreal Injection

ATX (100 μM, 1.5 μL; Sigma-Aldrich Co., LLC., St. Louis, MO, USA) dissolved in PBS was intravitreally injected under a microscope using a 33-gauge needle connected to an Ito Micro Syringe (Itou Corporation, Shizuoka, Japan) under anesthesia. PBS (1.5 μL) was intravitreally injected as the control. Retinal thickness at area 250 μm from the optic nerve head and the number of SRDs in each eye were evaluated using OCT. In experiments using the ATX inhibitor (HA130; 100 µM, 1.5 µL; Merck, Darmstadt, Germany), the inhibitor dissolved in DMSO or vehicle (DMSO alone; 1.5 µL) was intravitreally injected immediately after RVO induction.

### 4.5. Retinal Imaging

Fundus images within 60° of the posterior pole were obtained using a Canon CF-60DSI (Canon Inc., Tokyo, Japan) under systemic anesthesia as described above. Fluorescein angiography (FA) was then performed by intraperitoneal injection of 0.05 mL fluorescein (fluorescein 500 mg; Novartis International AG, Basel, Switzerland). Optical coherence tomography (OCT) images were obtained using SPECTRALIS (Heidelberg Engineering Inc., Heidelberg, Germany).

Whole retinal thickness was defined as the distance between the inner limiting membrane (ILM) and RPE. Retinal thickness of three random points, longer than 5 mm intervals from the optic disc and 1 mm from the center of laser spots toward the periphery area, was measured to avoid the potential thermal effect on retinal edema, and the average value was calculated.

### 4.6. Cell Culture and Transepithelial Electrical Resistance (TEER) Measurement

Primary human retinal microvascular endothelial cells (HRMECs; Cell Systems, Kirkland, WA, USA) were cultured using the Complete Medium Kit with serum and CultureBoost-R™ (Cell Systems). Primary human umbilical vein endothelial cells (HUVECs; American Type Culture Collection, ATCC, Manassas, VA, USA) were cultured in Vascular Cell Basal Medium (ATCC) supplemented with an Endothelial Cell Growth Kit (ATCC). Cells were incubated at 37 °C in a humidified atmosphere with 5% CO_2_. The culture medium was changed every 2 days until the cells reached confluence.

HRMECs and HUVECs (5.0 × 10^4^ cells) were seeded in 12-well Transwell plates (Corning Inc., New York, NY, USA) and cultured until confluence. ATX (0 nM, 10 nM, or 100 nM) dissolved in PBS was added to both the upper and lower compartments of the wells. TEER was measured at 1, 3, 6, 9, 24 and 48 h after treatment using a Millicell ERS-2 volt-ohm meter (Merck Millipore, Burlington, MA, USA).

To examine the effect of the ATX inhibitor, HA130 (100 nM) or vehicle (DMSO) was added at 1% volume to the culture medium in both chambers 30 min before ATX treatment. TEER values of cells were measured after 24 h of incubation with ATX. TEER values were calculated according to a previous report and are presented in Ω/cm^2^ [46].

### 4.7. Quantitative Polymerase Chain Reaction (qPCR)

Total RNA was isolated from HRMECs and retina using TRIzol reagent (Molecular Research Center, Cincinnati, OH, USA), according to the manufacturer’s instructions. cDNA was synthesized using ReverTra Ace qPCR RT Master Mix with gDNA Remover (Toyobo, Osaka, Japan). Quantitative real-time PCR was performed using TB Premix Ex Taq II (Tli RNaseH Plus; TaKaRa BIO, Inc., Shiga, Japan) and the Thermal Cycler Dice Real Time System III (TaKaRa BIO, Inc.). Relative mRNA expression levels were normalized to the housekeeping gene of β-actin. Primer sequences are listed in Table 1.

### 4.8. Statistical Analysis

All statistical analyses were performed using R software (R version 4.0.3; R Foundation for Statistical Computing, Vienna, Austria). Data are presented as mean values with standard deviations. The Wilcoxon rank-sum test was used to compare two groups. For comparisons among multiple groups, one-way or two-way ANOVA was performed, followed by Dunnett, Tukey–Kramer, or Bonferroni post hoc test. *p*-values < 0.05 were considered statistically significant.

## Figures and Tables

**Table 1:** DNA primers used for qPCR.

Oligos	Forward (5′–3′)	Reverse (3′–5′)
Human *MCP-1*	AGAATCACCAGCAGCAAGTGTCC	TCCTGAACCCACTTCTGCTTGG
Human *VCAM-1*	GATTCTGTGCCCACAGTAAGGC	TGGTCACAGAGCCACCTTCTTG
Human *IL-8*	ATGACTTCCAAGCTGGCCGTGGCT	TCTCAGCCCTCTTCAAAAACTTCTC
Human *VEGF-A*	CCCTGATGAGATCGAGTACATCT	AGCAAGGCCCACAGGGATTT
Human *β-actin*	CACCATTGGCAATGAGCGGTTC	AGGTCTTTGCGGATGTCCACGT
Mouse *ICAM-1*	CAATTTCTCATGCCGCACAG	AGCTGGAAGATCGAAAGTCCG
Mouse *VCAM-1*	GCTATGAGGATGGAAGACTCTGG	ACTTGTGCAGCCACCTGAGATC
Mouse *TNF-α*	GCCTCTTCTCATTCCTGCTTG	CTGATGAGAGGGAGGCCATT
Mouse *IL-1β*	GCCACCTTTTGACAGTGATGAG	TTAGGAAGACACAGATTCCATG
Mouse *IL-8*	ATGACTTCCAAGCTGGCCGTGGC	TCTCAGCCCTCTTCAAAAACTTC
Mouse *β-actin*	CATTGCTGACAGGATGCAGAAGG	TGCTGGAAGGTGGACAGTGAGG

MCP-1, Monocyte Chemotactic Protein-1; VCAM-1, Vascular cell adhesion molecule 1; IL, interleukin; VEGF-A, vascular endothelial growth factor-A; ICAM-1, intercellular adhesion molecule-1; VCAM-1, vascular cell adhesion molecule-1; TNF-α, tumor necrosis factor-α.

## Data Availability

The data supporting this study are available from the corresponding author (R.T.) upon reasonable request.

## Supplementary Materials

The following supporting information can be downloaded at: https://www.mdpi.com/article/10.3390/ijms27062776/s1.
