# Supplementary material for: Role of Autotaxin in the Pathogenesis of Retina Ischemia and Its Therapeutic Implications
Source: Int J Mol Sci. 2026 Mar 19;27(6):2776. doi: 10.3390/ijms27062776 (PMC13027173; doi:10.3390/ijms27062776)
Supplement: Supplementary file 1 [file ijms-27-02776-s001.zip › ijms-4108901-supplementary.pdf]

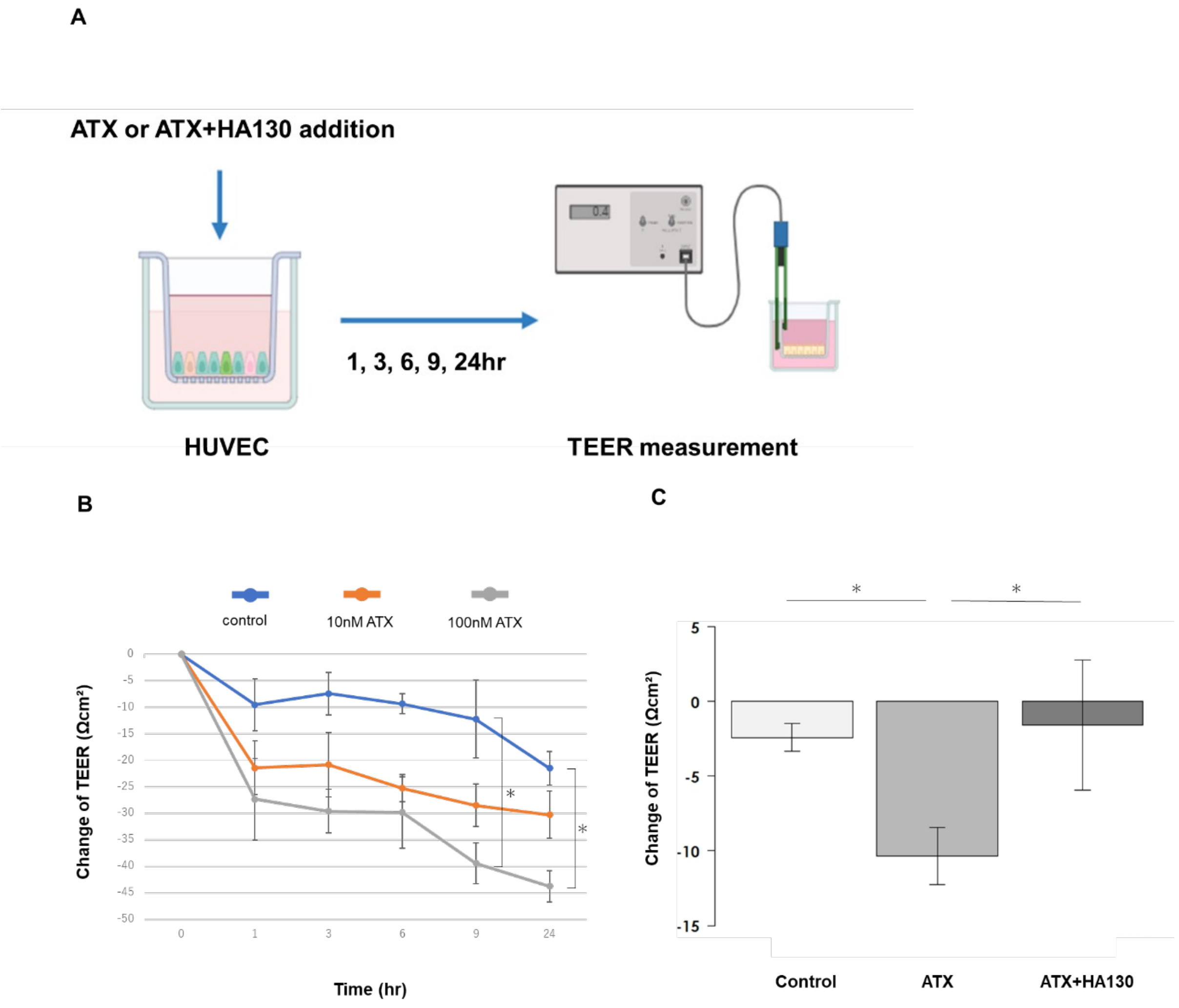

Supplementary Figure S1. Autotaxin disrupts barrier function of HUVECs
(A) Experimental design of transepithelial electrical resistance (TEER) measurement in human umbilical vein endothelial cells (HUVECs) treated with ATX and/or HA130. (B) Changes over time in TEER of HUVECs treated with ATX (100 nM or 10 nM) or PBS (Control). N = 4/group; *$p < 0.05$, Dunnett test; error bars are shown as standard deviations. (C) Effects of the ATX inhibitor (HA130; 100 nM) and ATX (100 nM) on TEER. n = 4/group; ＊$p < 0.05$, Tukey–Kramer test; error bars are shown as standard deviations. ATX, autotaxin; PBS, phosphate-buffered saline; HUVEC, human umbilical vein endothelial cells; TEER, transepithelial electrical resistance.
